# A Low-Cost 3-in-1 3D Printer as a Tool for the Fabrication of Flow-Through Channels of Microfluidic Systems

**DOI:** 10.3390/mi12080947

**Published:** 2021-08-11

**Authors:** Thana Thaweskulchai, Albert Schulte

**Affiliations:** School of Biomolecular Science and Engineering, Vidyasirimedhi Institute of Science and Technology, Rayong 21210, Thailand; thana.t_s17@vistec.ac.th

**Keywords:** microfluidics, microchannels, 3-in-1 3D printer, 3D printing, CNC milling, laser engraving

## Abstract

Recently published studies have shown that microfluidic devices fabricated by in-house three-dimensional (3D) printing, computer numerical control (CNC) milling and laser engraving have a good quality of performance. The 3-in-1 3D printers, desktop machines that integrate the three primary functions in a single user-friendly set-up are now available for computer-controlled adaptable surface processing, for less than USD 1000. Here, we demonstrate that 3-in-1 3D printer-based micromachining is an effective strategy for creating microfluidic devices and an easier and more economical alternative to, for instance, conventional photolithography. Our aim was to produce plastic microfluidic chips with engraved microchannel structures or micro-structured plastic molds for casting polydimethylsiloxane (PDMS) chips with microchannel imprints. The reproducability and accuracy of fabrication of microfluidic chips with straight, crossed line and Y-shaped microchannel designs were assessed and their microfluidic performance checked by liquid stream tests. All three fabrication methods of the 3-in-1 3D printer produced functional microchannel devices with adequate solution flow. Accordingly, 3-in-1 3D printers are recommended as cheap, accessible and user-friendly tools that can be operated with minimal training and little starting knowledge to successfully fabricate basic microfluidic devices that are suitable for educational work or rapid prototyping.

## 1. Introduction

As an advanced technological platform, the fabrication of microfluidic systems has relied on modern photo- and soft-lithography as the routine procedure [1]. While this method is still the gold standard for microfluidic device production, it is not the most accessible approach, as time-consuming work in costly cleanroom facilities and a high level of technical skill are required for the successful creation of miniaturized devices to precise microscopic specifications. In response to this drawback, alternative methods have been exploited for fabricating microfluidic device components, with the aim of enabling more research units and educational institutions to undertake synthetic and analytical microfluidic work. The transfer of the manufacturing process from cleanroom to desktop facilitated, for instance, the involvement of innovative strategies of microfluidic channel and pillar creation using instruments that are available in stationery or hardware stores and sold for other purposes. Tactics already tried include xurography [2], paper-based preparations [3,4], three-dimensional (3D) printing [5,6,7,8], computer numerical control (CNC) micro-milling [5,9,10,11,12] and laser engraving [12], many of which have found application in biomedicine [13,14], biosensing [15,16,17], bioprocessing [18], point-of-care (POC) diagnosis [19,20,21], industrial biotechnology [22] and other industries.

In recent years, instruments have appeared on the market with the functions of three-dimensional fused deposition modelling (FDM) printing, CNC milling and laser cutting/engraving integrated in easily switchable fashion. These so-called 3-in-1 3D printers operate by sharing a common workstation and *x*-, *y*-, and *z*-axis linear rails for movement of modular tool heads that can be swapped between functional units for 3D printing, CNC milling and laser cutting. Nowadays, 3-in-1 3D printers are available from several suppliers who offer a wide selection of consumer-friendly desktop versions targeted for home or office operation by users with little or no training in basic operation. Depending on their technical specifications, 3-in-1 3D printers vary in price and included features, and currently cost from just USD 600 for the simplest to USD 3600 for high-end models [23].

Individually applied in separate setups, 3D printing, CNC micro-milling and laser engraving have been tested in low-cost basic or in more expensive, technically advanced devices in fabricating microfluidic devices and shown to be viable alternatives to conventional photolithography [24,25,26,27,28,29,30,31]. CO_2_ laser-based fabrication of microfluidic substrates has, for example, been performed with common materials such as glass and poly methylmethacrylate (PMMA) sheets and the influence of the laser settings and the length and strength of surface treatments on the production of optimal microchannel profiles and on surface smoothness was demonstrated [32,33]. Applications of laser-fabricated microfluidic devices include droplet digital polymerase chain reaction (PCR) [34], droplet generation [35], wicking speed modulation on paper microfluidic devices [36] and culturing of embryonic bodies [37]. Feasibility trials have also been conducted with standalone devices for CNC milling [5] and 3D FDM printing [38,39,40,41], with satisfactory results. So far, however, to the best of our knowledge no multi-function 3-in-1 3D printer has been tested as a workstation for microfluidic device fabrication. We therefore investigated whether a reasonably priced commercial 3-in-1 3D printer could be a viable and affordable desktop option for microfluidic microchannel fabrication, particularly in rapid prototyping and/or production of basic microfluidic systems for research or educational use. The quality of completed chip plates with microchannels of different design and the time and cost of their production were evaluated. To ensure that the entire microstructure chip preparation and chip evaluation are not applicable only to specialized laboratory settings, the procedures were limited to basic precursor materials and standard laboratory instruments.

## 2. Materials and Methods

### 2.1. General Information

The 3-in-1 3D printer used in this study was the Snapmaker 2.0 Modular 3-in-1 3D Printer A250 (Shenzhen, China) with the bundled software Luban version 3.14.0 for control of 3D printing, CNC milling and laser operations (www.snapmaker.com, accessed on 15 February 2021). The 3D printing module has a nozzle diameter of 0.4 mm and a polylactic acid (PLA) filament of diameter 1.75 mm was used for the tests. The laser module unit has a 1600 mW laser diode of wavelength 450 nm and is classified in Safety Class 4. CNC milling worked with flat-end mill bits of various diameters. Polydimethylsiloxane (PDMS) chip plates were prepared with the Sylgard 184 silicone elastomer kit from Dow Chemical Company (Midland, MI, USA). Polymethylmethacrylate (PMMA) sheets of various sizes and thicknesses were used for micro-structured plastic chip and mold preparation.

3D microfluidic channel models were created as Computer-aided design (CAD) models using Autodesk Fusion 360 (Mill Valley, CA, USA) software. Model files were exported as standard tessellation language (STL) files for 3D printing and (computer-aided manufacturing) CAM files for CNC milling. Inkscape software (version 0.92.4) (Boston, MA, USA) was used to create vector files of microfluidic designs for laser engraving. The design files were exported as portable network graphics (PNG) or joint photographic group (jpg) files for use with Snapmaker-Luban (Shenzhen, China) software.

### 2.2. Microfluidic Channel Fabrication Using 3D Printing, CNC Milling and Laser Engraving

**Figure 1:**
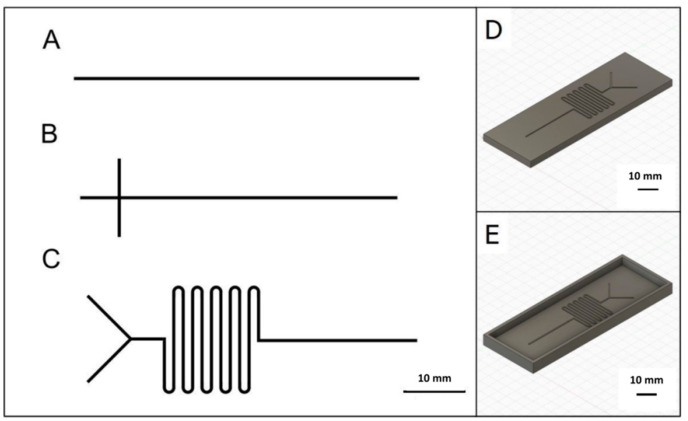
Microfluidic designs with 0.5 mm microchannel width and 0.15 mm microchannel depth. (**A**) Straight microchannel, (**B**) crossed junction microchannel, (**C**) Serpentine mixer microchannel. 3D rendering of (**D**) a microfluidic chip and (**E**) a microfluidic mold. The three designs were fabricated via three-dimensional fused deposition modelling (3D FDM), computer numerical control (CNC) milling and laser engraving.

To enable direct comparison, the microfluidic channel specifications were identical for the three types of fabrication (Figure 1). The microchannel geometries tested were a single straight channel, a crossed junction channel and a serpentine ‘mixer’ channel and their lateral extension was chosen to fit a standard microscope glass slide (75 mm × 25 mm). The smallest mill bit diameter available was 0.5 mm. Since the mill bit diameter controls the width of the microchannels produced as positive features by CNC milling, the default minimum microchannel width was 0.5 mm while the default depth was set to 0.15 mm.

### 2.3. 3D FDM Printing

All 3D FDM printing operations were controlled by the customized Snapmaker Luban software of the chosen 3-in-1 3D printer. Prior to printing, auto bed-leveling and print nozzle depth calibration were performed. The printing was then performed directly on PMMA print beds. Simultaneously, rectangular walls surrounding each design were formed at the edges of the print beds, to create the boundary for later PDMS casting. The parameter settings for the 3D FDM print procedure that formed the desired molds with line, crossed line and serpentine channel blueprints are outlined in Table 1.

Viscous mixtures of the PDMS silicon elastomer and curing agent were prepared and poured into finished molds on print beds still sited on the processing platform of the 3-in-1 3D printer. The PDMS could then be cured, by setting the print bed temperature control function to 50 °C. Once the PDMS was cured, the formed 75 mm × 25 mm rectangular silicon plate with integrated microchannel was carefully removed from the mold. PDMS plates with integrated microchannels obtained by 3D printing are hereafter referred to as ‘M-3D FDM’ chips. For flow tests, the PDMS plate was sealed with a PMMA cover plate of the same size, and the assembly fixed with a custom microfluidic chip holder to create the functional microfluidic device.

### 2.4. CNC Milling

The capability of the CNC milling option of the 3-in-1 3D printer in microfluidic device fabrication was assessed by (1) direct milling of the desired channel feature into pre-cut PMMA plastic plates and (2) fabrication of an elevated rectangular ridge structure and subsequent stamping of this target mirror feature into PDMS to create channels. To test option (1), the microfluidic target was directly milled with the rotating 0.5 mm drill bits into a 3 mm thick tailored PMMA sheet to create a shallow groove that can function as a microchannel. Following literature recommendations [33], key operational parameters were a spindle speed of 12,000 rpm, cutting feed rate 300 mm/min and plunge rate 36 mm/min. Directly milled PMMA chips with microchannels, hereafter referred to as ‘D-CNC’ chips, were assembled in the custom chip holder with equal-sized flat PDMS sheets to form the functional microfluidic unit. The blank chip cover mold with suitable side lengths and walls but no integrated 3D surface for the creation of PDMS counterparts was milled into a properly dimensioned PMMA plate, in an extra CNC step with identical parameter settings.

To test option (2), negative (ridge) fabrication and subsequent PDMS casting, a fitted 6 mm thick sheet of PMMA was used to fabricate the requisite microfluidic mold as a first step. Through choice of its border wall height, the mold was designed with a depth of 3.5 mm with the negative (ridge) feature located at the bottom, which allowed PDMS casting using conventional soft lithography. Finished PDMS plates with integrated microchannels are hereafter referred to as ‘M-CNC’ chips. For functional tests completed M-CNC PDMS chips were sealed with equally sized solid PMMA substrates and assembled to form a functional unit, using a custom chip holder. Based on the literature recommendations above [33], the operational settings used for milling depended on the actual mill bit diameter used. The details are discussed in the next section.

### 2.5. Laser Engraving

Laser-induced microchannel engraving was accomplished by importing the design files in png or jpg format into the Snapmaker Luban software, which then controlled the optical surface manipulation. The parameter settings for the laser engraving procedure that formed the desired microchannels with line, crossed line and serpentine channels are outlined in Table 2. Due to the specific type of the laser of the 3-in-1 3D printer used here, the PMMA targets had to be black in color for successful channel engraving. Like straight CNC milling of channel features, the laser directly engraved shallow grooves of one of the chosen microfluidic designs into the PMMA surface, but without physical contact. The fabricated units, from now on referred to as ‘D-Laser’ chips, were covered with a flat sheet of PDMS and assembled into a functional unit using the custom chip holder for flow measurements.

### 2.6. Soft Lithography

For all the above-mentioned PDMS casting steps the PDMS resin and curing agent were thoroughly mixed at a 10:1 ratio and the viscous blend was briefly degassed in desiccator connected to a vacuum pump. Immediately after preparation, the mixture was poured into the mold and, if not on the 3-in-1 3D printer platform, cured in an oven until hardened. The formed soft PDMS plate was then removed from the mold and, if required, inlet/outlet holes, to act as fluid access points, were made with a biopsy punch.

### 2.7. Optical Microchannel Inspection

The dimensions of the microchannels of completed microfluidic M-3D FDM, D-CNC, M-CNC and M-Laser chips were measured to evaluate the depth and width values of the created microstructures against the planned design specifications. To test positive feature fabrication of CNC milling and laser engraving, a straight channel with the width and depth of the designed microchannel was produced across the width of PMMA sheets. To test negative features, the mold with the test microchannel was produced first, then PDMS casting was performed to obtain the final channel in a PDMS plate. A Zeiss Microscope with an Axiocam 105 color digital microscope camera (Jena, Germany) was used to capture digital images of microchannel profiles and surfaces. Zeiss Zen 2.3 Lite software was used to record images and scale bars were added for further dimension analysis. Digital photographs of the cross-sections of test units were imported into ImageJ software (version 1.8.0_172) (Bethesda, MD, USA) for measurements of microchannel dimensions.

### 2.8. Functional Flow Tests

To confirm the feasibility of the technical strategies for functional flow channel fabrication, all produced microfluidic devices underwent flow testing. This involved infusing the microfluidic device, an assembly of a chip plate with and a chip plate without a microchannel, with liquid colored either green or red for enhanced fluid visualization and observation of the flow behavior. Infusion at a flow rate of 50 µL/min was controlled by a syringe pump (PHD Ultra Syringe Pump, Harvard Apparatus) (Holliston, MA, USA). One criterion of the flow test was that the liquid flow in the microfluidic device was restricted to the microchannel, with no leakage along its length.

## 3. Results and Discussion

As shown in Figure 1, the three microchannel layouts selected for the 3-in-1 3D printer tests in this study were a single straight line, a crossed junction and a serpentine mixer, each of which was used to test different microfluidic features. The straight-line design was simplest to fabricate and represented the most fundamental and widely used microchannel type. The crossed junction design was an ideal pattern for testing the creation of more complex microfluidic channel types with liquid flow in channels that meet at a right-angle and then continue as one. The serpentine microchannel with a Y-shaped junction on the inlet side for input of two different solutions and a meandering path enabled tests of channel designs tailored for effective liquid mixing during passage. Demonstration of feasible fabrication for the three chosen targets offered confirmation that a cheap, consumer-grade 3-in-1 3D printer with a small bench footprint is a useful tool for constructing microfluidic channel structures, in general and particularly for laboratories with space and/or budgetary constraints.

**Figure 2:**
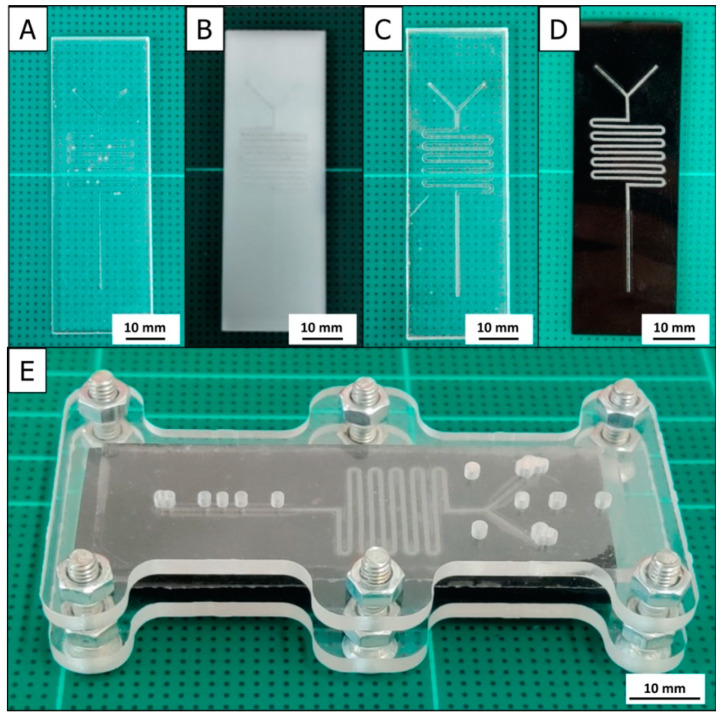
Photographs of a 3D FDM-printed polydimethylsiloxane (PDMS) (**A**), a D-CNC-milled white PMMA (**B**), a M-CNC-molded PDMS (**C**), and a laser-engraved microfluidic black PMMA chip plate (**D**) all with serpentine flow channel integration. Shown in (**E**) is the assembly of a chip plate, here a laser-engraved PMMA microchannel chip plate with a PDMS lid plate in an adapted plate holder.

Simple visual checks and microscopic inspection with digital photography of entire fabricated microfluidic chip plates with channel structures revealed that all four methods successfully reproduced the planned designs, with no obvious imperfections. Figure 2 shows a laser-engraved serpentine microchannel on a black PMMA base plate, as a representative example of the final product of 3-in-1 3D printer-based microfabrication. Additionally shown is the assembly of such a microchannel base plate with the matching PDMS lid to form the desired microfluidic chip. Closer optical inspections of structural fine details such as dimensional accuracy and conformity to two-dimensional (2D)- and 3D-profiles are discussed in the following sections.

### 3.1. Specific Remarks on M-3D FDM Chip Fabrication

Microfluidic device fabrication using 3D FDM printing involved directly printing the desired microchannel features onto the plastic sheet print bed using molten PLA polymer filament as the starting material. For good results with 3D printing, strong adherence of the first printed layer was of the utmost importance as it defined the strength of the bonding of the entire 3D structure to the print bed. Guided by relevant literature, a rather slow print speed of 9 mm/s was therefore selected. The required height of the first printed layer was 0.15 mm. This matched the expected microchannel depth and accordingly allowed the printing machine to complete the necessary channel mold feature in a single scan across the entire print bed. Subsequent print layers were needed to form the mold walls at the edge of the PMMA plate, and a greater layer height and faster print speed were therefore used, to reduce the total printing time. Once the printing process was complete, the PDMS mixture could conveniently be introduced into the printed trough with the microchannel mirror structure and held there at 50 °C until cured.

3D printing with PLA filaments and the 3-in-1 3D printer device of this study was a very straightforward way to form molds for microfluidic PDMS channel casting. Once the procedure has been established it operates as a single programmed step with virtually no risk of user error adversely affecting the quality of the product. This is particularly valuable for applicants with little or no experience in 3D printing or microfabrication in general, as it is the simplest method and has a high chance of success. The process of mold fabrication required only a cheap 3-in-1 3D printer and no additional instrument or tool, except for a PDMS kit as material.

It is worth mentioning that acrylonitrile butadiene styrene (ABS) and nylon are two other commonly used thermoplastic 3D printing materials and recently the PLA, ABS and nylon set has been expanded by, for instance, filled nylon, a composite of the polymer with stronger materials such as fiber glass or carbon fibers [42]. This proof-of-principle study was restricted to PLA 3D printing. Potential applicants of the proposed methodology are encouraged, on the other hand, to test microchannel fabrication in a low-cost 3-in-1 3D printer device with polymer filaments other than PLA as, after achieving adequate practical skills and experience with optimal process parameter setting, the chance of useful adaptations of customized microchannel fabrication is increased.

### 3.2. Specific Remarks on Milled CNC Chip Fabrication

The final quality of CNC milled workpieces depends on the parameters used during the milling process. The factors determining the choice of the diameter of the mill bits were the microchannel dimensions and geometry. For positive features, where the microchannel was directly milled on the PMMA substrates, the milling bit diameter was 0.5 mm since this was the width of the microchannel. For CNC fabrication of master molds for PDMS microchannel chips with line or crossed line designs, mill bits of diameter of 1 mm were used, the increased tool diameter offering a balance between the requirement for fine detail milling, especially at corners, and the minimization of total milling time, as larger diameter bits complete the process of surface abrasion across the entire board in shorter time. To mill the master molds for the serpentine microchannels the inner diameter of the semi-circle turn was 1 mm and an 0.8 mm diameter mill bit was better for this task. The spindle speed was 12,000 rpm for both mill bit sizes. For the 0.8 mm diameter mill bit, the cutting feed rate was 700 mm/min, the plunge feed rate 40 mm/min and the optimal load 0.048 mm. For milling with the 1 mm mill bit, the corresponding settings were 900 mm/min, 40 mm/min and 0.06 mm.

### 3.3. Specific Remarks on Laser-Engraved Chip Fabrication

Laser engraving is commonly seen as the ideal process for speedy microchannel prototyping. Target channel patterns can be designed with readily available tailored graphic design software such as the Autodesk Fusion 360 package used here, and prepared layout files can be uploaded into the laser system and used for direct microfluidic channel carving into polymer plate loads in just a few minutes. The work speed and fixed power percentage were important procedure parameters here as they dictated the level of laser energy delivered to the black PMMA substrates to form the channel structure. Settings of 500 mm/min and 50% permitted channel processing to the targeted depth of 0.15 mm.

### 3.4. Comparison of Obtained Microchannel Cross-Sections and Surfaces

**Figure 3:**
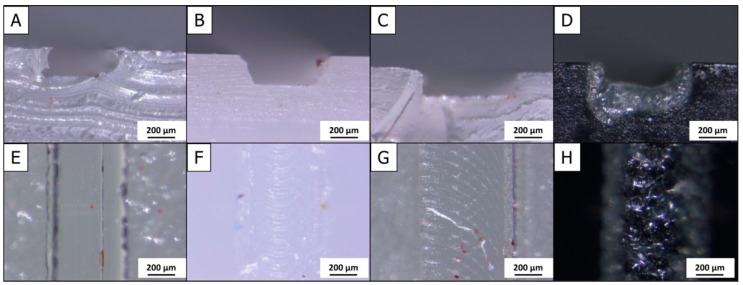
Microscopic pictures of cross-section profiles and surface appearances of microchannels as prepared with the tools of a 3-in-1 3D printer device. Cross-sections (**A**–**D**) and surfaces (**E**–**H**) are of (**A**)/(**E**) a 3D FDM-, (**B**)/(**F**) a D-CNC-, (**C**)/(**G**) a M-CNC-, and (**D**)/(**H**) a laser-fabricated microchannel.

Figure 3 shows photographs of cross-sectional profiles and surfaces of representative examples of M-3D FDM-, D-CNC-, M-CNC- and D-Laser-produced microchannels. To examine PDMS microchannels obtained with molds from the 3D FDM and M-CNC fabrication procedures, it was necessary to cut across the rubber material to expose the channel’s cross-sectional profile. It is evident from the micrograph of a 3D FDM microchannel cross-section in Figure 3A that transections, carried out in the 3-in-1 3D printer device by CNC micro-milling, had the tendency to deform the channel structure, leaving the vertical side walls and flat bottom distorted rather than straight. This was, however, an effect of the sample preparation rather than an accurate depiction of the channel’s quality. Usually the surface of the bottom of 3D FDM microchannels appeared uniform and level, with only minor surface streaks along the channel length and the channel walls were accurately parallel (Figure 3E).

The cross-section profile of D-CNC microchannels appeared trapezoid in shape, with the top of the channel slightly wider than the bottom (Figure 2B). The cause of this characteristic profile is likely to be quivering of the mill bits that carved the structure. At initial contact this lateral movement is stronger but is dampened as the bit moves deeper into the material, resulting in the observed truncated cone channel profile. Looking at the bottom surface of the CNC-milled microchannel (Figure 2F), a row of wavy marks was observed along the length of the microchannel left by the mill bits as they traversed the surface. Adaption of the CNC milling parameters would, in principle, allow minimization of this kind of surface roughness of carved channels; however, the delicacy of very thin mill bits, with the attendant risk of tool fracture, limited the choice of operating parameters and the quality shown in Figure 3B,F was the best possible with the D-CNC microchannel milling in the 3-in-1 3D printer used in this study.

M-CNC microchannels that were cast in PDMS using a milled M-CNC mold showed a rectangular cross-sectional profile with straight vertical side-walls and a flat bottom (Figure 2C). The surface of molded M-CNC PDMS microchannels showed a pattern of semi-circular markings, similar to that seen on the D-CNC chip surface (Figure 2G). However, the radius of the semi-circles in M-CNC PDMS items was larger, due to the use of a thicker mill-bit for CNC mold fabrication.

Laser engraving of black PMMA base plates produced microchannels with cross-sections and surfaces as shown in Figure 2D,H, respectively. Both the profiles and surface appeared rather rough and irregular, indicating variabilities of laser-induced material ablation across and along the microchannel structure, resulting in surface discoloration and bump formation. The surface quality of laser-engraved microchannels was obviously lower than that of equivalent pieces made by M-3D FDM-, D-CNC- or M-CNC machining, which produced microchannels with walls and bottom surfaces that were generally flat and even, with only minor surface imperfections, which favors suitable flow dynamics during practical applications. The rough surfaces of laser-carved microchannels, on the other hand, may not be optimal for controlled laminar flow at the contact region between the moving liquid and inner pathway walls.

### 3.5. Comparison of Feasible Microchannel Dimension Accuracy

Table 3 lists all the straight versions of four microchannel types used in this study, with their measured depths and widths and the dimensional accuracy achieved, as a percentage of the microchannel dimensions of 0.15 mm depth and 0.5 mm width.

The microfabricated chip that conformed best to the required microchannel depth and width produced was created by a combination of a mold fabrication by 3D printing and subsequent PDMS casting for channel formation (the ‘M-3D FDM’ chip). Apparently, the 3D printer resolution was adequate to duplicate the specified dimensions with errors of <10% in the mold structure finally used for channeled chip formation. The 0.5 mm diameter mill bits were used to carve microchannels 0.5 mm wide and 0.15 mm deep straight into solid PMMA plates to produce ‘D-CNC’ chips, and the procedure did reasonably well in producing the required channel width (deviation <10%) but showed a poorer performance in the reproduction of the channel depth (deviation ≈ 44%). To save time, milling of the PMMA molds for ‘M-CNC’ microfluidic chip fabrication was carried out with larger (1 mm diameter) mill bits, a modification that led to an about 30% reduction of the width accuracy but improved the channel depth reproduction by about 25%, compared to direct microchannel carving. As expected from microscopic inspections, the microchannels of D-Laser microfluidic chips showed major deviations from the target dimensions, channel depth and width differing from the ideal values by about 54 and 20%, respectively.

The achievable depth accuracy of microchannel fabrication depends both on the inherent specifications of the printing machine and on factors that are independent of the quality of the commercial microfabrication device. Manual tool tip pre-positioning just above the surface, for instance, is required prior to mill bit- or laser-driven surface shaping and thus uniformity of the thickness of the PMMA sheets influences reproduction of the correct channel depth. Inspections of PMMA precursor sheets with a digital vernier caliper revealed thickness variation of up to 0.05 mm, an imprecision that adds to device-related errors. Taking this into account, and with the positive outcome of the flow tests in Section 3.3, the performance of the cheap 3-in-1 3D printing machine for PMMA and PDMS microchannel preparation was acceptable, certainly for the purpose of basic technology research, early feasibility studies and educational introduction to microfluidics, although last-stage system development and commercial launch and operation would obviously require higher grade, more costly commercial devices.

### 3.6. Functional Microfluidic Channel Flow Tests

**Figure 4:**
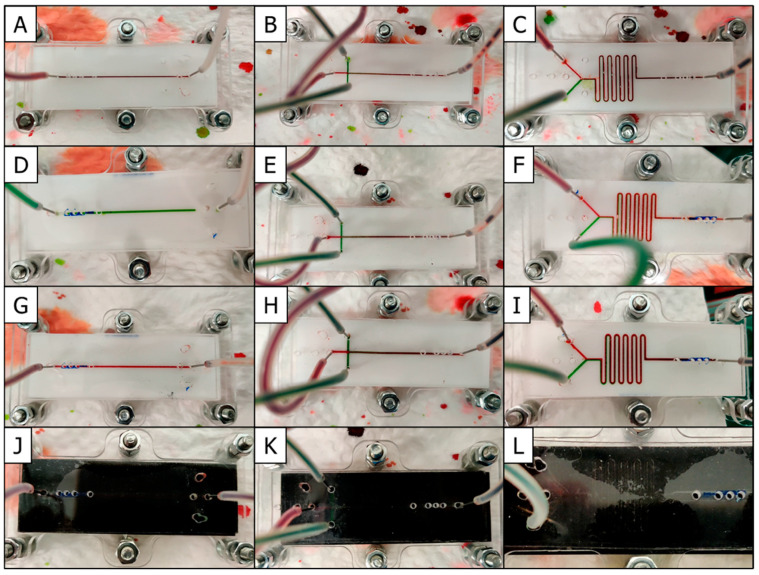
Photographs of microfluidics devices from various fabrication methods and designs being tested under colored liquid flow at 50 µL/min. (**A**) M-3D FDM with single straight channel, (**B**) M-3D FDM with crossed junction, (**C**) M-3D FDM with mixer channel, (**D**) D-CNC with single straight channel, (**E**) D-CNC with crossed junction, (**F**) D-CNC with mixer channel, (**G**) M-CNC with single straight channel, (**H**) M-CNC with crossed junction, (**I**) M-CNC with mixer channel, (**J**) Laser Chip with single straight channel, (**K**) Laser Chip with crossed junction, and (**L**) Laser Chip with mixer channel.

To accomplish the microchannel flow tests, a particular unit was joined with an equal-sized solid PMMA substrate (for D-CNC and D-Laser chip plates) or a soft PDMS sheet (for the D-3D FDM chip plates) and the sandwich secured with the specially designed assembly holder (see Figure 2). For easy visualization of liquid movement from inlet to outlet, the aqueous solution pumped through the channels was tinted with either red or green food coloring. The dynamics of the flow tests were videotaped in real time and in addition snapshots of each test were taken (Figure 4). All three types of fabrication method led to practical microfluidic devices through which sample fluids streamed without leakage. The Y-shaped serpentine mixer microfluidic chip plate showed two discrete streams of dyed fluids approaching each other at the junction before complete passage through the entire meandering portion of the microchannel led ultimately to their fusion and formation of a uniform, single-colored stream. For microfluidic chips with the crossed junction microchannel design, three streams of fluid merged at their intersection to initially form distinct bands of colored zones before gradually mixing into a single homogenous flow. This basic function of fluid mixing is crucial in microfluidic devices as it allows some of the most common tasks to be carried out within the chip, including fluid dilutions and mixing, the starting or stopping of chemical reactions, droplet creation and sample preparations, to mention just a few. The visualization of the flow tests confirmed that all four fabrication methods created practical microfluidic platforms.

**Figure 5:**
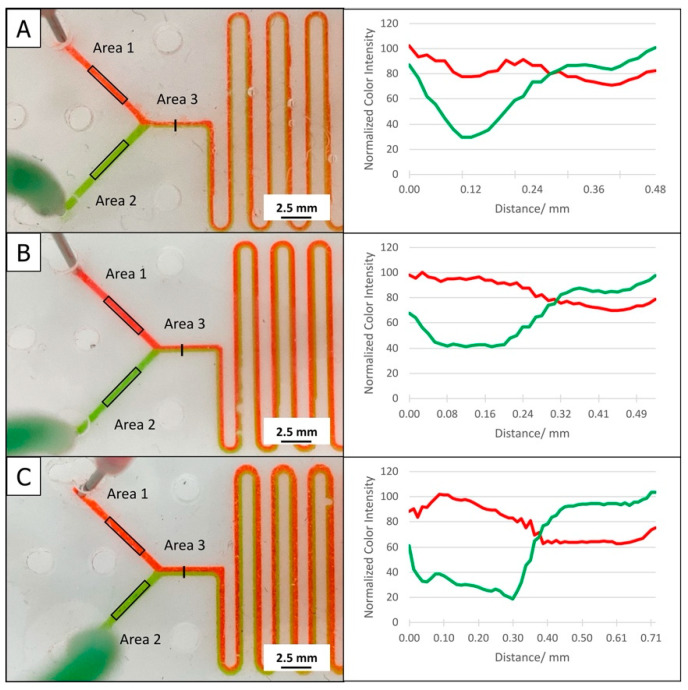
Close-up photographs of assembled microfluidic device with the serpentine mixer microchannel fabricated by (**A**) D-3D FDM, (**B**) D-CNC, and (**C**) M-CNC. The three device configurations were tested under colored liquid flow at 50 µL/min Laminar flow of the two fluids is obvious as they merge at the Y-junctions. The graphs to the right show cross-sectional profiles of the color intensity at area 3. The profiles were constructed with the normalized color intensities, calculated by taking the color intensity value in area 3 divided by baseline color intensity from area 1 (red) or area 2 (green) multiplied by 100 and plotted against the location in the channel (top wall considered to be 0).

To demonstrate the influence of channel roughness and the “v-shaped” cross section on the flow regime, an optical RGB color profile analysis with dyed input fluid streams at the Y-junction was performed using close-up photographs of the serpentine microfluidic devices fabricated by M-3D FDM, D-CNC, and M-CNC (Figure 5). Microfluidic channels fabricated by the D-Laser procedure were excluded from the inspection due to poor channel visibility on the dark chip plates. The flow rate for the trial was kept for the two inlets at 50 μL/min, a typical value for microfluidic applications. Measurements of the red, green blue (RGB) color intensity were made at three specific locations along the microchannel using ImageJ software. Regions 1 and 2 were occupied by bare red and green fluid, respectively, and the color intensity measurements across the channel width were used as baseline level. At region 3, a cross-sectional profile of the RGB color intensity of the merged streams of colored fluids was taken and expressed as normalized color intensity over distance across the microchannel width. Normalized color intensity was calculated by taking the color intensity value in region 3 divided by baseline color intensity from region 1 (red) or region 2 (green) multiplied by 100. Looking at the close-up photographs, all three devices demonstrated two distinct streams of colored fluids after merging at the Y-junction, which continued in the serpentine mixing section, just as, for instance, was observed in a previous study by others for the fusion of three colored streams in a three-inlet microchip [29]. There are, however, some differences between the three fabrication methods that can be observed. For the M-3D FDM channel in Figure 5A, the interface of the two fluids was not as distinct and sharp as for the two channels from CNC fabrication. The graph representing the color intensity profile also suggested that there was slight mixing of the fluids. This may be due to irregularities in microchannel geometries, combined with the textured surface of the 3D printer print bed that caused minute leakage, resulting in unintended mixing of the two colors. In contrast, the channels fabricated by M-CNC showed the highest contrast in color intensity as confirmed by the cross-sectional profile of color intensity (Figure 5C). This observation may relate to a microchannel geometry that was better defined and wider, compared to the other two options (refer to Figure 3 and Table 3). This produced improved laminar flow, especially along the microchannel walls, and the wider microchannel improved smooth fluid flow towards the channel middle, resulting in higher color intensity of individual colors and better contrast while the two colors merged.

### 3.7. Comparison of Feasible Fabrication Time

Depending on the choice of the fabrication procedure, the time required to complete a microchannel chip varied from just a few minutes to several hours. 3D printing of the mold for M-3D FDM PDMS chip preparation took, for instance, approximately 15 min while the subsequent casting of the mold structure into rubber-like PDMS required up to 4 h for the curing of the viscous silicon precursor/catalyst blend load. Speedier curing at an elevated temperature was not possible because of the low glass transition temperature of the printed PLA material (50–80 °C) that formed the mold structure and walls, with a consequent risk of deformation. The creation of positive microchannel features through direct CNC milling (D-CNC chips) or laser engraving (D-Laser chips) of PMMA substrates was obviously much quicker and functional chip completion was possible within 2–5 min per chip unit, depending on the complexity of the microchannel design, e.g., the choice of simple straight or more demanding serpentine geometry. CNC milling of a mold for M-CNC chip preparation lasted about 2 h, since except for the elevated narrow negative microchannel structure and the mold walls, a large amount of material of the PMMA plate had to be removed by milling using a mill bit of relatively small diameter. As the transition temperature of PMMA glass is higher than that of PLA, the requisite curing of the viscous silicon/catalyst PDMS blend in a finished CNC mold could be accomplished in a convection oven at 70 °C in less than 1 h, a few-fold faster than for the molding step of the 3D printing process. A shared advantage of using 3D-printed or CNC-milled molds for microchannel chip fabrication is that multiple copies can be made through repeated use of the same template.

### 3.8. Cost/Benefit Analysis

The cost of equipment and complexity of the procedure are barriers to the wide- spread use of microfluidic device technology in the analytical and science programs in high schools, technical colleges and universities, certainly in developing countries. The desktop 3-in-1 3D printer used in this study for microfluidic chip manufacture is offered online for USD 1499, excluding shipping and tax. So far, instruments for fabrication of microfluidic devices usually had considerably higher costs. Terminals with desktop CNC micro-milling machines may, for instance, cost several thousand USD for basic versions or up to USD 100 k for high-end devices [10,11,41]. The prices of laser engraving stations depend on the power and quality of the laser optics and sophisticated desktop versions of heavy-duty CO_2_ lasers for microchannel fabrication cost a few thousand USD or more, depending on the device specification [43]. Obviously, more highly priced systems will have the advantage of advanced technical specifications, and they may, for instance, incorporate a compact system enclosure, machine cabinet air filtering and cabinet and drilling stage temperature control, automatic tool change (ATC), auto-bed leveling and calibration, multiple nozzle printing, multiple axis milling, and hard- and software adaptations, permitting more complex 3D printing and micro-milling operations and the reduction of overall fabrication time. It is, however, unlikely that this class of machine will be widely adopted, because of their cost. However, cheap (USD < 1000) single-function 3D- or 3D-FDM printers and CNC and laser micro-milling machines are available nowadays, and on the market for USD 1000–2000 as 3-in-1 multi-function combinations. A benefit of using a slightly more expensive 3-in-1 micromachining tool is the consequent simplification of workflow and device operation through a bundled software, which reduces the learning process for beginners. For educational purposes and entrance level microfluidic research and development (R&D) microchannel fabrication through micro-printing and -milling a 3-in-1 3D printer device, as used this study, is an ideal compromise between economy and practicality.

### 3.9. Limitations

While the 3-in-1 3D printer device tested in this study has proved to be capable of the fabrication of basic functional microfluidic devices using a variety of methods, there are some shortcomings to be addressed. First, the CNC module of the system is limited to the milling of polymer chip plates and cannot process harder materials such as glass and metals. Glass, on the other hand, is often the preferred substrate material of a microfluidic platform and is commonly used, for instance when biological cells from primary or secondary cell culture and tissue engineering are studied within the microchannel space. Second, the laser module, with a 1.6 W laser diode and 450 nm wavelength, has no effect on laser engraving of transparent or white PMMA. Nevertheless, the use of transparent microfluidic devices has numerous advantages, including the ease of visualization during flow operations and the chance to integrate optical sensing and advanced microscopy as analytical methodologies. Finally, the three fabrication methods, namely 3D printing, CNC milling and laser engraving, require manual inputs from the user as part of their execution procedures. Though the user involvement is not demanding, and the amount of training needed is minimal for each method, procedures are only semi-automatic, and neatness and attention are essential for successful fabrication of microfluidic devices to specification.

## 4. Conclusions

To date, this is the first study to explore the possibility of using a low-priced 3-in-1 3D printer for microfluidic device fabrication, with individual use of all three available functions—3D printing, CNC milling and laser engraving—for microchannel chip and base-plate formation. Evidence is provided that functional microfluidics can be accomplished with varying degrees of success with all three functions. The availability of three micromachining strategies in one cheap workstation has advantages and demands compromises. Table 4 summarizes the main advantages and disadvantages of the three integrated functionalities of the 3-in-1 3D printer device used in this study and also condenses the benefits and drawbacks of their joint presence in one affordable workstation. The cost is reasonable even for underfunded laboratories and the requirement for bench space is minimal. Its operation requires minimal skills and training is quick and easy, enabling new users to produce microfluidic chips independently within a short period of time. While there are certain limitations, e.g., the minimum size of microfluidic channel features that can be fabricated, the customized machine can function as an affordable microfabrication station for basic microfluidic designs in educational settings to introduce tangible, functional examples of microfluidics to new students or for rapid prototyping of novel microfluidic designs in academic research laboratories.

As modern 3-in-1 3D printers continue to be developed, with the release of newer and improved models in various price ranges, these machines have the potential to act as an access point to microfluidics technology and to reach demographics that would otherwise be unable to use this technique. It is worth mentioning that we recently reported a simple and low-cost DIY procedure for the fabrication of a planar three electrode chip platform that, when combined with common straight PDMS microchannels, performed well in microfluidic voltammetry, amperometry and electrochemical enzyme biosensing [58]. Application of microchannels as prepared here in combination with the analytical DIY electrode chips of the previous study has a great potential for the realization of user-friendly, low-cost but effective microfluidic electroanalysis without budget restrictions.

## Figures and Tables

**Table 1:** 3D fused deposition modelling (FDM) printing parameters used in microfluidic channel mold fabrication.

First Print Layer Height	0.15 mm	Wall Thickness	0.8 mm
First layer print speed	9 mm/s	Nozzle initial temperature	205 °C
Following layer height	0.24 mm	Nozzle print temperature	200 °C
Outer wall speed	20 mm/s	Infill	0%
Inner wall speed	25 mm/s	Print bed temperature initial layer	70 °C
Top/bottom speed	30 mm/s	Print bed temperature	50 °C

**Table 2:** Parameters used for laser engraving of microfluidic channel structures into black poly methylmethacrylate (PMMA).

Laser Power	1.6 W	Laser Wavelength	450 nm
Processing mode	Vector	Fill density/power	20/50%
Jog speed	1000 mm/min	Work speed	500 mm/min

**Table 3:** Statistics on the depths and widths of 3D FDM, D-CNC, M-CNC and laser-engraved straight microchannels as fabricated in a low-priced 3-in-1 3D printer device. Each value is the average of determinations at three cross-sections of a representative example of the microchannel chip plates. The depth and width % values were calculated as actually measured dimension divided by the desired dimension multiplied by 100.

Chip Type	Depth (mm)	Depth (%)	Width (mm)	Width (%)
M-3D FDM	0.162 ± 0.009	108.4 ± 5.9	0.515 ± 0.017	102.9 ± 3.5
D-CNC	0.216 ± 0.004	143.8 ± 2.8	0.539 ± 0.081	107.9 ± 16.0
M-CNC	0.174 ± 0.008	116.2 ± 5.1	0.690 ± 0.006	138.0 ± 1.1
D-Laser	0.231 ± 0.043	153.8 ± 28.9	0.600 ± 0.011	119.9± 2.2

**Table 4:** Advantages and disadvantages of the fabrication of microfluidic microchannel fabrication via 3D printing, CNC milling and laser ablation and the benefits and drawbacks of the joint availability of the toolset in a low-cost 3-in-1 3D printer device.

Method	Advantage	Disadvantage	References	This Study
				3-in-1 3D Printer Device
3D printing	Low cost Rapid operation Shape flexibility	Limitation to available printing filaments	[43,44,45,46,47,48,49,50]	Low cost Simplicity Small bench footprint Ideal starter kit Elementary functionalities Limitation in resolution Weak laser component
CNC milling	Low cost Material flexibility	Strong surface impact Surface roughness Shape restriction	[10,43,50,51,52,53]	
Laser ablation	Rapid operation Good precision	Limited material choice Surface roughness	[43,50,54,55,56,57]	
